# Decision-analytic modeling for early health technology assessment of medical devices – a scoping review

**DOI:** 10.3205/000313

**Published:** 2022-12-21

**Authors:** Annette Conrads-Frank, Petra Schnell-Inderst, Silke Neusser, Lára R. Hallsson, Igor Stojkov, Silke Siebert, Felicitas Kühne, Beate Jahn, Uwe Siebert, Gabi Sroczynski

**Affiliations:** 1 Institute of Public Health, Medical Decision Making and Health Technology Assessment, Department of Public Health, Health Services Research and Health Technology Assessment, UMIT TIROL – University for Health Sciences and Technology, Hall i. T., Austria; 2 Alfried Krupp von Bohlen and Halbach Foundation Endowed Chair for Medicine Management, University of Duisburg-Essen, Essen, Germany; 3 Center for Health Decision Science, Departments of Epidemiology and Health Policy & Management, Harvard T. H. Chan School of Public Health, Boston, MA, USA; 4 Institute for Technology Assessment and Department of Radiology, Massachusetts General Hospital, Harvard Medical School, Boston, MA, USA; 5 Division of Health Technology Assessment, ONCOTYROL – Center for Personalized Cancer Medicine, Innsbruck, Austria

**Keywords:** early health technology assessment, medical devices, decision-analytic modeling, cost-effectiveness analysis

## Abstract

**Objective:**

The goal of this review was to identify decision-analytic modeling studies in early health technology assessments (HTA) of high-risk medical devices, published over the last three years, and to provide a systematic overview of model purposes and characteristics. Additionally, the aim was to describe recent developments in modeling techniques.

**Methods:**

For this scoping review, we performed a systematic literature search in PubMed and Embase including studies published in English or German. The search code consisted of terms describing early health technology assessment and terms for decision-analytic models. In abstract and full-text screening, studies were excluded that were not modeling studies for a high-risk medical device or an in-vitro diagnostic test. The Preferred Reporting Items for Systematic Reviews and Meta-Analyses (PRISMA) flow diagram was used to report on the search and exclusion of studies. For all included studies, study purpose, framework and model characteristics were extracted and reported in systematic evidence tables and a narrative summary.

**Results:**

Out of 206 identified studies, 19 studies were included in the review. Studies were either conducted for hypothetical devices or for existing devices after they were already available on the market. No study extrapolated technical data from early development stages to estimate potential value of devices in development. All studies except one included cost as an outcome. Two studies were budget impact analyses. Most studies aimed at adoption and reimbursement decisions. The majority of studies were on in-vitro diagnostic tests for personalized and targeted medicine. A timed automata model, to our knowledge a model type new to HTA, was tested by one study. It describes the agents in a clinical pathway in separate models and, by allowing for interaction between the models, can reflect complex individual clinical pathways and dynamic system interactions. Not all sources of uncertainty for in-vitro tests were explicitly modeled. Elicitation of expert knowledge and judgement was used for substitution of missing empirical data. Analysis of uncertainty was the most valuable strength of decision-analytic models in early HTA, but no model applied sensitivity analysis to optimize the test positivity cutoff with regard to the benefit-harm balance or cost-effectiveness. Value-of-information analysis was rarely performed. No information was found on the use of causal inference methods for estimation of effect parameters from observational data.

**Conclusion:**

Our review provides an overview of the purposes and model characteristics of nineteen recent early evaluation studies on medical devices. The review shows the growing importance of personalized interventions and confirms previously published recommendations for careful modeling of uncertainties surrounding diagnostic devices and for increased use of value-of-information analysis. Timed automata may be a model type worth exploring further in HTA. In addition, we recommend to extend the application of sensitivity analysis to optimize positivity criteria for in-vitro tests with regard to benefit-harm or cost-effectiveness. We emphasize the importance of causal inference methods when estimating effect parameters from observational data.

## Zusammenfassung

**Zielsetzung:**

Unser Ziel war es, entscheidungsanalytische Studien zur frühen Technologiebewertung von Medizinprodukten aus den letzten drei Jahren zu identifizieren und einen systematischen Überblick über den Zweck der Modelle und die Modellcharakteristika sowie über aktuelle Entwicklungen in der Modellierungstechnik zu geben.

**Methoden:**

Für diesen Scoping Review wurde eine systematische Literatursuche in den Datenbanken PubMed und Embase nach Studien in englischer und deutscher Sprache durchgeführt. Der Suchcode setzte sich aus Begriffen für die frühe Technologiebewertung und Begriffen für ein entscheidungsanalytisches Modell zusammen. Im Screening von Zusammenfassung und Volltext wurden Studien ausgeschlossen, bei denen es sich nicht um ein Modell für Hochrisiko-Medizinprodukte oder diagnostische Tests handelte. Für alle eingeschlossenen Studien wurden Zweck und Rahmen der Studie sowie Modellcharakteristika extrahiert und zusammenfassend in sytematischen Evidenztabellen sowie in narrativer Form dargestellt.

**Ergebnisse:**

Aus 206 Studien wurden neunzehn Studien in den Review eingeschlossen. Die Studien betrafen entweder hypothetische Medizinprodukte oder existierende Produkte, nachdem sie bereits auf dem Markt erhältlich waren. Keine Studie nutzte die Extrapolation technischer Daten aus frühen Entwicklungsstufen, um den potentiellen Wert für die Gesellschaft zu evaluieren. Mit Ausnahme einer Studie schlossen alle Studien Kosten in die Evaluation ein. Zwei Studien waren Budget-Impakt-Analysen. Die meisten Studien zielten auf die Aufnahme in den Leistungskatalog und die Kostenerstattung der Medizinprodukte ab. Die Mehrheit der Studien betraf In-Vitro-Diagnostika für personalisierte und zielgerichtete Interventionen. Zeitgesteuerte Zustandsautomaten, ein Modelltyp, der bisher nicht im HTA genutzt wurde, wurden zur Beschreibung von komplexen, individuellen klinischen Pfaden und von Interaktionen dynamischer Systeme eingesetzt. Nicht alle Unsicherheitsquellen bei In-vitro-Tests wurden explizit modelliert. Das Einholen von Expertenwissen und Expertenbeurteilungen wurde als Ersatz für fehlende empirische Daten verwendet. Die Analyse der Unsicherheiten stellte den größten Vorteil der entscheidungsanalytischen Modellierung im frühen HTA dar, aber kein Modell wandte Sensitivitätsanalysen an, um den Schwellenwert für Testpositivität hinsichtlich der Nutzen-Schaden-Abwägung oder der Kosteneffektivität zu optimieren. Die Value-of-Information-Analyse wurde selten eingesetzt. Die Anwendung von Methoden der kausalen Inferenz zur Schätzung von Effektparametern aus Beobachtungsstudien wurde in keiner Studie erwähnt.

**Schlussfolgerung:**

Unser Review gibt einen Überblick über Ziele und Modelleigenschaften von neunzehn Studien zur frühen Bewertung von Medizinprodukten. Der Review zeigt die wachsende Bedeutung der personalisierten Medizin und bestätigt frühere Empfehlungen der sorgfältigen Modellierung der mit diagnostischen Tests verbundenen Unsicherheiten und eines vermehrten Einsatzes der Value-of-Information-Analyse. Zeitgesteuerte Zustandsautomaten könnten eine lohnenswerte Erweiterung der Modelltypen im Rahmen der Technologiebewertung sein. Zusätzlich empfehlen wir die Anwendung von Sensitivitätsanalysen zur Optimierung des Schwellenwerts für Testpositivität hinsichtlich der Nutzen-Schaden-Relation oder der Kosteneffektivität. Wir betonen die Wichtigkeit der Anwendung von Methoden der kausalen Inferenz für die Schätzung von Effektparametern aus Beobachtungsdaten.

## Introduction and background

Medical devices comprise a multitude of heterogeneous products with about 500,000 different medical devices currently being available on the European market. Mainly medical devices of high-risk classes, classes IIb and III (for example implants) according to the European Union Medical Device Directives [1], [2], [3], Medical Device Regulation and classes C and D of the European Union In-vitro Diagnostics Regulation [4], [5], respectively, are subject to health technology assessment to inform health care decision makers, primarily for reimbursement and coverage decisions [6]. The International Network of Agencies for Health Technology Assessment (INAHTA) and Health Technology Assessment International (HTAi) have developed a new definition of health technology assessment (HTA) in 2020: “HTA is a multidisciplinary process that uses explicit methods to determine the value of a health technology at different points in its lifecycle. The purpose is to inform decision-making in order to promote an equitable, efficient, and high-quality health system” [7]. The different points in the lifecycle are described as “pre-market, during market approval, post-market, through to the disinvestment of a health technology” [7].

A lifecycle approach to HTA with repeated assessments at time points dependent on the kind of decision that has to be made (e.g. investment in research and development, market approval, reimbursement and coverage), and from the perspective of the relevant decision makers (manufacturer, regulatory agency, HTA bodies, provider) is especially relevant to medical devices. For medical devices, innovation is characterized by short product life cycles, a process of incremental development, and often insufficient evidence to assess clinical effectiveness and cost-effectiveness at time of licensing [8].

The specific challenges and recommendations for gathering evidence, comparative effectiveness research and HTA of devices for reimbursement and coverage decisions, including coverage with evidence development schemes, have been studied extensively in recent years [9], [10], [11], [12], [13], [14], [15]. Processes and methods of HTA agencies to evaluate medical devices have been described as well [16], [17]. Not all countries evaluate systematically cost-effectiveness in HTA. For example Germany, France and many Southern European countries only assess the added clinical benefit, but not cost-effectiveness in their HTA reports for decision-making bodies [18]. Besides the fast and incremental development, the effectiveness and cost-effectiveness of medical devices is often dependent on contextual factors such as skills and experience of providers, infrastructure and organization [11], [14]. This is not unique to medical devices, but relevant especially to high-risk devices such as implants that data on long-term effectiveness and safety accrue only over time, mainly in registries. Further, specific issues to be considered for economic evaluation of medical devices are dynamic pricing, and partially also high upfront cost and capital investments (e.g. computed tomography scanner). Dynamic pricing in the field of medical devices is often characterized by a decrease of prices due to short product cycles and quick market entry of competitors [19], [20]. For example, empirical evidence shows a considerable decrease in prices for drug-eluting stents between 2006 and 2014 in several European countries and the US. But there are other devices such as single-chamber pacemakers where prices kept stable or even increased in some countries [20].

Besides HTA for reimbursement decisions in the post-market phase, early and repeated assessment has long been recommended for innovative technologies to guide investment into research and development [21], [22], [23], [24], [25], [26].

Systematic reviews on methods in early HTA in general and in early HTA for medical devices have been performed within the last 12 years [22], [27], [28], [29], [30], [31]. The latest of these reviews, by IJzerman et al. in 2017, defines early HTA as “all methods used to inform industry and other stakeholders about the potential value of new medical products in development, including methods to quantify and manage uncertainty” and identified five main reasons for conducting early assessments of research and development strategies: preclinical market assessment, portfolio decisions, clinical trial design, and market access and pricing strategies [28]. With regard to evidence generation in trial phases, Sculpher et al. [21] located “early HTA” in a phase when evidence for clinical effects is typically available from small uncontrolled case series, that is, a time point when first clinical evidence from phase I and II clinical trials but none from RCTs is available. It has been argued for medical devices that data from technical studies, in-vitro and animal studies or safety studies may allow for an assessment already in the development phase of a technology, and it has been suggested that even in the conceptual stage, the potential maximum incremental effectiveness and cost of a new technology may be assessed [21].

The most frequently used methodology in early HTA is health economic modeling [28]. In health economic modeling, a decision-analytic model is used to compare a new technology with established comparators considering effectiveness and costs [32], [33]. Decision-analytic models can integrate evidence of different types of studies such as clinical trials and epidemiologic studies, combine evidence from studies on diagnostic accuracy and from efficacy trials for subsequent treatment, take into account patient preferences and allow for evaluation of uncertainty and for estimation of the value of additional research [34]. They can also be helpful in the design of trials. Models can be adapted relatively easily to reassess a product after modification or after new data are becoming available. This is important for keeping up with the fast pace of innovation in medical devices. On the other hand, especially in early HTA, modeling needs to deal with gaps in empirical data. Methods for elicitation of expert knowledge have been developed to address this problem in decision-analytic models [35], [36], [37]. Good practice guidelines are available for development and reporting of decision-analytic modeling studies.

In their review on early HTA, IJzerman et al. [28] found studies applying traditional modeling techniques like decision trees and Markov models, but two studies employed different techniques to incorporate dynamic interactions in the health care system and future changes in the application of a medical product. The authors of the review foresee future modeling needs in reflecting dynamic interactions and in describing complex clinical pathways with sequential and often personalized testing and treatment.

The goal of this systematic review was to identify recently published decision-analytic modeling studies in early health technology assessment of high-risk medical devices and provide an overview of model purposes and modeling techniques.

This review included the following specific research questions: (a) In which stages of development of a medical device were the modeling studies performed and for what purpose are the devices assessed?, (b) Why have decision-analytic models been developed and what are the strengths of decision-analytic models that the studies exploited?, and (c) Are there new developments in decision-analytic modeling for early HTA of devices since the review published in 2017 by IJzerman et al. [28]?

## Methods

We performed a systematic literature search in Medline via PubMed and in Embase to identify decision-analytic models in early health technology assessments of high-risk medical devices. For the search, keywords for “early HTA” and keywords for “decision-analytic model” were combined by a logical “AND”. The exact search code is reported in Attachment 1 (Table 3, Table 4). The search was restricted to publication dates from January 1, 2017 to April 17, 2020. Publications before this date were already covered by the review by IJzerman et al. [28]. We also limited our search to publications with available German- or English-language full text.

Abstract and full-text screening based on a priori defined inclusion and exclusion criteria was performed by one author (ACF) and confirmed by a second author (PSI). Publications were excluded if

1. the study did not perform a health technology assessment, a benefit-harm assessment, a budget impact analysis or a health economic evaluation,
2. the assessment was not performed at an early stage in device development, defined as a stage where the new technology was not yet widely adopted and where data from large randomized controlled trials (RCT) were not yet available (this definition corresponds to phases one and two in the categorization by Sculpher et al. [21]),
3. the assessed technology was neither a high-risk medical device nor an in-vitro diagnostic test, or
4. the study was not a decision-analytic modeling study.

One author (ACF) extracted the following characteristics of the modeling studies: reference, study type, device name or type, phase of development, purpose of the study, population, intervention, comparator, outcomes, model type, available evidence for the effect of the device, model assumptions about the effect that distinguishes the new device from existing devices, data sources for cost, evaluation of uncertainty, performance of a value-of-information analysis, employment of expert elicitation.

Results are provided in systematic evidence tables and narrative, descriptive result summaries for therapeutic and for diagnostic devices. The narrative result summaries are structured to reflect the purpose of the studies and the use of decision-analytic methods (model structure, data use, uncertainty evaluation).

Since the goal of this systematic review is a scoping exercise of the literature published between 2017 and 2020 and not a synthesis of study results, we did not exclude studies based on quality [38].

## Results

**Figure 1:**
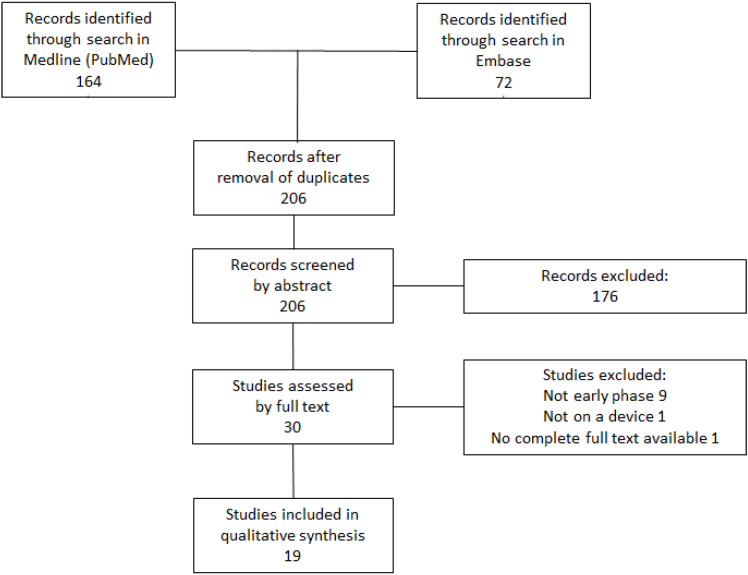
PRISMA diagram for the selection of studies

The systematic literature search resulted in 206 studies after exclusion of duplicates. After abstract and full-text screening, 19 studies were included in the review (Figure 1 (Fig. 1)). Since devices do not have a value per se, it was strictly not the devices that were evaluated, but interventions involving these devices. The evaluated devices were therefore often a general type of device, not necessarily a specific approved product on the market. Many intervention strategies had two components, such as a test and a medical treatment.

We identified four studies on therapeutic interventions [39], [40], [41], [42]. Among those, two were for assessing implantable devices, a customized knee implant [40] and a device for aspiration therapy [39]. Two were for assessing new MRI-assisted surgical interventions, laser interstitial thermal therapy for epilepsy [42] and pulmonary vein isolation for atrial fibrillation [41]. Overall, we found 15 studies on diagnostic devices [35], [43], [44], [45], [46], [47], [48], [49], [50], [51], [52], [53], [54], [55], [56]. One of these studies assessed an invasive device, a single-use bronchoscope [54]. The other 14 studies evaluated interventions involving in-vitro tests [35], [43], [44], [45], [46], [47], [48], [49], [50], [51], [52], [53], [55], [56]. Table 1 (Tab. 1) gives an overview of the types of in-vitro tests and their area of application.

### Therapeutic devices

Details of extraction results for therapeutic devices are given in Table 2 (Tab. 2) and in Attachment 1 (Table 5). The following section summarizes results relevant to our research question.

#### Stage of development and purpose of the study

The purpose of the study by Wenker et al. [41] was to evaluate whether investment in the idea of a new MRI-guided intervention for atrial fibrillation would have a chance to result in a cost-effective new procedure. The authors point out significant technical challenges, which still need to be overcome to realize their idea. For Mital and Nguyen [39], the purpose of the study was to evaluate the potential market for the aspiration therapy device. The study assesses the cost-effectiveness of the intervention and aims at finding the target population for which the technology is cost-effective. The MRI-guided laser intervention for epilepsy assessed by Widjaja et al. [42] is already performed in practice despite lack of RCT data and without an HTA. The purpose of the study is to fill the gap in assessment with an emphasis on the uncertainty surrounding data input. The authors see their analysis as the first step in an iterative process of health technology assessment. The goal of the study is an analysis of the effectiveness and cost-effectiveness of the intervention and estimation of the value of future research through a value-of-information analysis. The purpose of the study by Namin et al. [40] for a custom knee prosthesis was to promote wider adoption of the product. The long-term cost saving quality of their product was already established and the product was reimbursed by payers. The goal of the study was the evaluation of potential savings for the health care payer with wider adoption of the product under more generous reimbursement schemes than the current.

Wenker et al. [41] assessed a hypothetical procedure, while the other three interventions were already performed in clinical practice but not adopted widely yet. It was not clear if the studies were initiated by the manufacturers or by public health assessment authorities [39], [40], [41], [42].

#### Data

No experimental clinical data were available for any of the interventions. In the hypothetical study by Wenker et al. [41], the main effect parameter was an assumption and was varied in a wide range. For Namin et al. [40] and Widjaja et al. [42], effect parameters came from a single retrospective study each, of 235 and 234 patients, respectively. Mital and Nguyen [39] used data of 200 patients from a post-market registry.

All modeling studies were performed from the perspective of the health-care payer. Indirect costs were not considered. Cost data for the new products were estimated from product prices and investment costs provided by the manufacturer and were partially derived from comparisons to similar procedures and costs for similar clinical consequences.

#### Modeling approach

The model for a hypothetical surgical procedure for atrial fibrillation [41] was a decision tree with a one-year time horizon, evaluated in cohort simulation. Mital and Nguyen [39] used a Markov cohort model to simulate the effects of weight loss on mortality over the lifetime of patients. Widjaja et al. [42] developed a lifetime microsimulation state transition model to describe complex patient pathways including potential subsequent procedures after the initial MRI-guided thermal therapy and to calculate lifetime costs using detailed resource use and cost data from a patient-level costing study. Namin et al. [40] developed a systems dynamics model for an eight-year time horizon. The authors argue that the new customized knee replacement is beneficial for patients and saves costs in the long run for the payer but has higher upfront costs so that current reimbursement rates, which are the same as those for the traditional knee replacement, prevent clinical adoption by hospitals. The systems approach was chosen because it allows for modeling of reimbursement schemes, surgeon and patient decisions and clinical outcomes, including the feedback loops between these entities. The authors also wanted to report population-level costs over time and therefore also included modeling of the number of procedures over time in the target population. Parameters for this subsection of the model were calibrated and validated by comparison to historical data. For none of the other models a calibration or validation procedure was performed. The time horizon of eight years for the model by Namin et al. [40] was sufficient to show longer-term savings of the new intervention. Three studies [40], [41], [42] discussed the importance of the learning curve for adoption of the evaluated technology, but no study explicitly modeled the effect.

#### Uncertainty

For all models, sensitivity analyses were an important part of the model results. Except for the hypothetical evaluation by Wenker et al. [41], all studies performed a probabilistic sensitivity analysis. Three models performed a range of deterministic sensitivity analyses [39], [41], [42]. Two models performed scenario analyses [40], [42] and the central task of the hypothetical model by Wenker et al. [41] was the threshold analysis for the effect of the intervention. Widjaja et al. [42] performed a value-of-information analysis and found considerable expected monetary benefit in performing additional clinical trials. Collecting information on event and progression probabilities after the new MRI-based technique was found to have higher value than collecting information on utilities. None of the other studies on therapeutic devices performed a value-of-information analysis.

### Diagnostic devices

Detailed results for diagnostic devices are shown in Attachment 1 (Table 6, Table 7).

#### Stage of development and purpose of the study

The purpose of studies on hypothetical tests was to explore under which conditions for test accuracy (sensitivity, specificity) and test price, biomarker-guided strategies would be cost-effective [43], [44], [48], [50], [51], [53] or effective [55]. For non-hypothetical tests, the purpose of the studies was to promote adoption of a product that was deemed to reduce adverse events [54], find target populations for which these strategies could be cost-effective [45], [49], to inform decisions on investment into a non-reimbursed product [49], to explore potential clinical strategies by eliciting expert opinion on clinical utility of a new test [35], or create a basis for incorporating biomarkers into clinical decision making [52]. For the study by Degeling et al. [46], the simulation served the purpose to evaluate an extension of the application area of circulating tumor cells from disease monitoring to response monitoring. Doble et al. [47] see their study as the beginning of value assessment in multiplex-targeted sequencing for advanced lung cancer treatment and recommend repeated future assessment as the technology develops and testing parameters improve. They also expect sequencing tests to expand the field of application from advanced lung cancer to other patient populations, for example testing at diagnosis or other cancers, generating further needs for adapting and developing their assessment in the future. The reason for the budget impact analysis of Yu et al. [56] was to inform reimbursement decisions for next generation sequencing tests.

While one [55] of the 15 studies [35], [43], [44], [45], [46], [47], [48], [49], [50], [51], [52], [53], [54], [55], [56], [57] assessed clinical effectiveness only, all other studies included cost outcomes. The majority of studies were cost-effectiveness or cost-utility studies. One study was a budget impact analysis [56].

The study on a single-use bronchoscope evaluated a device on the market [54]. Of the 14 studies on in-vitro diagnostics [35], [43], [44], [45], [46], [47], [48], [49], [50], [51], [52], [53], [55], [56], seven were hypothetical tests [43], [44], [48], [50], [51], [53], [55], although the authors of some of these studies envisioned certain types of tests: a DNA test [44], a biomarker assay [51], and a pharmacogenomics test [53]. Five studies evaluated already developed in-vitro tests [45], [46], [47], [49], [56], among those tests for single biomarkers [45], [49], a test for circulating tumor cells [46], and two next generation sequencing tests [47], [56]. One further study evaluated a hypothetical combination of three available single in-vitro biomarker tests [35] and one study evaluated a hypothetical single in-vitro biomarker test but used test characteristics from data on three different available single biomarker tests [52].

#### Modeling approach

Model types were decision trees in five studies [35], [43], [49], [52], [54] with time horizons between six hours for the test on myocardial infarction and five years for two studies in cancer. Markov models combined with a decision tree were developed in five studies [45], [47], [48], [50], [56]. Four further studies performed microsimulations. These models were combinations of decision trees with previously published state-transition models [44], [51] (cancer), a discrete event simulation [53] (cardiovascular disease, CVD) and a decision tree combined with a survival model [55] (cancer). The study by Degeling et al. [46] focuses on the comparison of two microsimulation model types, discrete event simulation and timed automata. The study includes modeling of repeated testing for treatment response monitoring and potential treatment switching to the next line treatment. Physician adherence to recommended testing intervals and treatment interruptions unrelated to progression were also modeled. While discrete event simulation has been applied less frequently than Markov models in health technology assessment, it is a well-established technique in HTA. Modeling with timed automata on the other hand is, to our knowledge, a novelty in health technology assessment. The timed automata model is a type of agent-based model. It consists of separate models for the agents and entities in the clinical pathway, in this case patients, physicians, tests and guidelines. Each of the models consists of a finite number of states and potential transitions between those states. Messages can be sent from one model to another and transitions occur due to incoming messages or time and may be subject to constraints. Which transition occurs may be probabilistic. The process can keep track of time in each state and of costs. Agents can act together or jointly. The discrete event simulation model on the other hand describes the system as a single process where individual patients experience a sequence of probabilistic events. The authors showed that both model types could represent the decision problem at hand and lead to similar results.

In the study by Terjesen et al. [54], only adverse effects were considered different between the assessed device and the comparator and modeling of test accuracy was not needed. Among the in-vitro diagnostic studies, test sensitivity and specificity constituted the main difference between comparators in the hypothetical studies (based on assumption and varied in sensitivity analysis), while one non-hypothetical study used response rate conditional on biomarker results (predictive values of response) [52], and one study used survival conditional on test positivity and targeted treatment [56]. Among the four non-hypothetical studies on personalization of treatment [46], [47], [52], [56], Degeling et al. [46] is an example of a study that included test sensitivity and specificity in their modeling for each repetition of the test. The study by Doble et al. [47] for a multiplex-targeted sequencing test included the widest range of sources for uncertainty surrounding the test and of consequences of the test itself: a) biopsy samples may be insufficient for testing, b) multiplex-targeted sequencing may not be successful, c) successful tests have limited accuracy (sensitivity, specificity), and d) if alterations are found, they may not be actionable, meaning that there may not be a targeted treatment proven to be effective with these alterations. The testing also takes considerable time, so the authors included mortality during the four-week testing phase and considered different starting times for the treatment options. Adverse events caused by the biopsy were also considered. Yu et al. [56] considered the reduction in unsuccessful tests for a next-generation sequencing test for lung cancer and assumed test accuracy for individual alterations the same for single marker tests and next-generation sequencing. Lotan et al. [52] considered the probability of test positivity and the probability of treatment response with positive test results. Of the remaining two models, Critselis et al. [45] included test accuracy in their model for a diagnostic test for kidney disease in diabetes. Khoudigian-Sinani et al. [49] presented the only model where the test result (risk of oral cancer) was a continuous risk that was not immediately dichotomized. This would have offered the opportunity to evaluate the risk cut-off point for resection of the lesion that leads to optimal effectiveness or cost-effectiveness. The authors chose a different approach by asking an expert panel at which risk their decision on subsequent medical treatment would likely change.

#### Data

In line with our inclusion criteria, no RCT data were available yet for the complete test-and-treat strategies in the included studies. The hypothetical studies either chose test accuracy to be the same as the comparator [44] or assumed values and varied them in sensitivity analyses [43], [44], [48], [50], [51], [53], [55]. Several studies did not refer to a specific device and manufacturer, but evaluated certain types of tests in general, examples of which are available on the market [46], [47], [49], [52], [56]. In Doble et al. [47] for example, test accuracy was modeled on the basis of a published study which reported on the sensitivity and specificity for detecting any genomic alteration measured by a general next-generation sequencing panel. The main treatment effect parameters, response to and mortality after targeted therapy and standard therapy, were taken from published studies on the average effect of targeted therapy for a range of different alterations. In Yu et al. [56], data were based on published literature about the already available single gene tests which were assumed to be included in the next-generation sequencing assay and on the established targeted treatment for two of these alterations. The model of Lotan et al. [52] combines data for test positivity and response with targeted treatment collected in first studies for three different biomarkers. Degeling et al. [46] estimated the accuracy of the association between circulating tumor cell count and treatment response in advanced lung cancer from one study on the relation between cell count and survival. The study of a three-biomarker test [35] calculated accuracies from the characteristics of the individual biomarkers included in the combined test. For the diagnostic model by Critselis et al. [45], data were available for diagnostic accuracy from one published study. In Khoudigian-Sinani et al. [49] the positive predictive value of the test was known from one empirical study, but the distribution of cases on different risk categories was based on assumptions.

Expert knowledge or opinion was used by several studies to inform the clinical utility of tests and define test-treatment strategies [35], [45], [49] or to fill model parameters for which data were missing [46], [50], [51], [54], [56]. Terjesen et al. [54], Khoudigian-Sinani et al. [49], and Kip et al. [35] used a formalized process for this purpose. Terjesen et al. [54] based the risk of infection after bronchoscopy with the established re-usable device on consensus estimates from a two-round Delphi survey among experts and on rates for the new single-use device on the assumption of zero infection risk. Khoudigian-Sinani et al. [49] drafted scenarios for potential strategies based on the test results of the new test and presented standardized questionnaires to a panel of four experts to elicit the beliefs about the impact of the new test on clinical management. Kip et al. [35] elicited the probability of discharge and follow-up diagnostics with the new test for myocardial infarction at different levels of accuracy for this test from 10 cardiologists in a detailed standardized questionnaire.

#### Uncertainty

All studies performed extensive sensitivity analyses, mostly deterministic analyses and scenario analyses. Lansdorp-Vogelaar et al. [51] is an example where an especially large array of scenario and sensitivity analyses was performed to assess a multitude of potential screening strategies and test accuracies. Six studies performed probabilistic sensitivity analyses [35], [43], [44], [48], [49], [50]. In four studies, threshold analyses were an important part of the assessment [48], [50], [51], [53]. Mitchell et al. [53] and Kluytmans et al. [50] for example varied sensitivity, specificity and the price of the device to find thresholds for cost-effectiveness. None of the studies employed sensitivity analysis to define the cut-off for an optimal positivity criterion of the test with regard to the benefit-harm relation or cost-effectiveness.

To inform future research, Doble et al. [47] performed a value-of-information analysis and calculated the expected value of perfect information (EVPI) and the expected value of partial perfect information (EVPPI) for groups of parameters (testing parameters, probabilities of state transitions for each of the three comparators, costs for each of the three comparators, and health state utility values) using a nonparametric regression-based method. The EVPI represents the expected value of conducting research to eliminate the uncertainty of all model parameters. The EVPPI represents the value of conducting research to eliminate the uncertainty for just some of the model parameters. Doble et al. [47] also estimated population EVPI and EVPPI in addition. The authors found considerable value for reducing uncertainty overall. They found the largest value in reducing uncertainty for cost and resource use parameters. No other study on testing devices performed a value-of-information analysis.

## Discussion

We performed a systematic scoping review on decision-analytic modeling in early HTA of high-risk medical devices. We focused on recent studies published between 2017 and 2020, to assess the new evidence after the publication of earlier reviews of similar kind. In line with current trends and predictions [22], the majority of the included studies focused on in-vitro diagnostics and personalized treatments based on these diagnostics. Most of them were applications in cancer, which is not surprising, as biopsy tissue is available to be analyzed for biomarkers associated with disease progression or treatment response.

### Stage of development and purpose of the study

Except for one recently developed test [45], all devices assessed in the included studies were either hypothetical or had already been made available on the market, but had not achieved wide-spread diffusion yet. Some of the devices had already been assessed for reimbursement by national health technology agencies and were either denied reimbursement or were reimbursed, but struggled for adoption nevertheless. Therefore, there were several examples of studies where promoting market diffusion was a reason for performing the assessment [39], [40], [49]. On the other hand, the study by Widjaja et al. [42] aimed at providing a critical assessment of an intervention with increasing market diffusion that had not been thoroughly evaluated yet.

In agreement with the review by IJzerman et al. [28], we found that decision-analytic models were developed to assess cost-effectiveness in almost all studies included in our review. Decision-analytic modeling allowed to combine effects of different test-treatment strategies on benefits, harms and cost, and therefore, assess the balance between benefits, harms and all costs. These are particularly important purposes of modeling in diagnostic test strategies, as discussed in a report of the Agency for Healthcare Research and Quality in the USA [58], [59] and in the report of the ISPOR Personalized Medicine Special Interest Group [60]. The modeling process forces the researcher to explicitly describe and quantify the target population, to compare strategies and all components of the clinical pathways, and to extrapolate to patient-relevant outcomes and costs for the intended audience. This leads to collection of data for population characteristics, disease progression probabilities, intervention effects, and resource utilization and can help to make gaps in data obvious. This process is helpful especially in early HTA where applications of new devices are explored.

### Reasons for decision-analytic modeling

In addition, decision-analytic modeling allows for estimation of outcomes under uncertainty. For all the early assessments found in this review, sensitivity analyses presented the main tool to derive insight on the potential value of new interventions. For many hypothetical interventions especially, ranges of device characteristics and device and intervention cost resulting in cost-effectiveness were the goal of the study. Deterministic one- and two-way analyses and threshold analyses were performed to find these ranges. On the other hand, none of the studies made use of the possibility to optimize the positivity criterion of a test, that is, the optimal cut-off point on the receiver-operating characteristic (ROC) curve. Studies therefore missed to fully optimize the tradeoff between the consequences of false negative (sensitivity) and false positive (specificity) test results, which strongly affect the benefit-harm relation and the cost-effectiveness ratio. In fact, this means that not all possible comparators were considered, which is a key principle in HTA [61]. Scenario analyses were frequently used in addition to other sensitivity analyses, allowing for assessing variations in structural model assumptions in addition to variations in parameter values. One study on a hypothetical screening test [51] performed a large number of scenario analyses and presented an example of the use of decision analysis to simulate a high number of different screening strategies, beyond a number possible to study in a clinical trial. A large proportion of studies also assessed the overall parameter uncertainty in probabilistic sensitivity analyses.

### Developments in decision-analytic modeling for early HTA

Previous reviews discussed the possibility of performing value-of-information analysis in early HTA on the basis of a decision-analytic model and recommended to include value-of-information modeling to assess the value of further research and to design further research studies in an optimal way [22], [27], [62]. Our review showed that this method is still not frequently used. Only two of the nineteen studies in our review performed a value-of-information analysis [42], [47]. Both studies could not only show the general value of future research, but also pointed out the group of parameters for which the greatest value of future research can be expected.

Since for the term “early HTA”, our review defined “early” as a stage where RCT data on the assessed interventions are not available yet and even the clinical application of a new device may not yet be clear, studies had to find a way to fill gaps in data. Elicitation of expert knowledge has frequently been mentioned as an appropriate source of information in the absence of empirical data [63], [64], and nearly half of the studies in our review mentioned consultation of experts in some way. Two studies stood out in applying a structured form for elicitation of expert beliefs or knowledge [35], [49]. In both cases, the authors were seeking input on the potential clinical application of a new test. Both drafted a number of scenarios for potential clinical actions after applying new tests in addition to established ones, and consulted an expert panel through a structured questionnaire. Terjesen et al. [54] described a two-stage Delphi panel method to obtain a consensus estimate for their central model parameter and its uncertainty. These three studies [35], [49], [54] did not refer to the reporting guidelines for elicitation studies published in 2016 [64], but all three reported in detail on the methods used.

Other studies used data from existing similar devices and interventions for an initial estimate of effects and cost. For a diagnostic multimarker test, data were derived for example from tests of the individual markers [35]. For an unspecific predictive single biomarker test, data from three different specific tests were combined for input on test characteristics [52]. Of course, the hypothetical studies often used mere assumptions and relied completely on the subsequent sensitivity analysis.

The review by IJzerman et al. [28] pointed to the study by Pietzsch et al. [30] as the first to introduce a systems engineering approach to support manufacturers in decisions about device development. This is a study where technical failure mode analysis provided the basis for an early decision-analytic assessment of effectiveness and cost of a device in the early stages of development. We did not find any study of this type in our review. The reason may be that decision-analytic modeling is usually not performed before approval, or it may be that this type of study is not published in the data bases that we searched. IJzerman et al. [28] assume a publication bias due to confidentiality and intellectual property rights.

For personalized, test-guided treatment, Rogowski et al. [65] pointed out the importance of modeling of uncertainties surrounding the test. In a report of the ONCOTYROL – Center for Personalized Cancer Medicine, Rogowski et al. [65] additionally explained the value of individual preferences in optimizing decisions for patients. Di Paolo et al. summarized that many of these challenges have not been overcome yet in personalized medicine [66]. Faulkner et al. [15], addressing value frameworks in precision medicine (using for example next-generation sequencing tests), pointed to the importance of considering test performance, penetrance, pathogenicity and linkage to patient management and outcomes. In our included early HTA studies, such levels of detail were rare. One study in our review [47] presents an exceptional example, where various sources of uncertainty were explicitly considered in the model. This study on fourth-line treatment of metastatic lung adenocarcinoma modeled the sources of failure in a next-generation sequencing test including insufficient biopsy samples, unsuccessful testing, negative effects for false positive results, the consequences of delays in treatment while waiting for test results, and the possibility that detected alterations are not actionable.

For personalized treatment approaches and dynamic system behavior, the review by IJzerman et al. [28] expected an increased need for dynamic and patient-level modeling. Regarding the model type and simulation approach, two studies in our review may present examples for arising model approaches. In a personalized treatment study on response monitoring with circulating tumor cells in prostate cancer, Degeling et al. [46] used two microsimulation model types, discrete event simulation and timed automata, to simulate the consequences based on complex individual patient histories, caused by repeated testing of treatment response and potential treatment switching. Both model types were able to simulate complex pathways of the decision problem and were therefore appropriate applications in personalized medicine. While timed automata have been used in modeling of technical real-time systems and networks, mainly in modeling of computer networks, for over 20 years, we were not aware of any application in health technology assessment. It may be a type of modeling approach worth exploring in the future in cases where timing is complex and the interaction of many agents is important. Another new development in the model approach was presented by Namin et al. [40]. The authors presented a system dynamics model, which is not a new modeling approach per se, but they included not only clinical outcomes and costs, but also modeling of reimbursement schemes, surgeon and patient decisions, and the feedback loops between all these entities. The focus of this study was on higher reimbursement, which was assumed to increase adoption of the new technology over time.

The majority of the studies included in our review used state-of-the-art modeling techniques that are frequently used in regular HTA reports for reimbursement applications at a later stage in evidence development. Many studies cited international modeling good practice guidelines [57], [67], [68], [69], [70], [71], [72] and in general adhered to the guidelines. We also found in our review that reporting was mostly transparent in the studies [72].

In line with the increasing role of real world data in health care policy [73], we found that three studies on available therapeutic devices (i.e., knee replacement, aspiration device, and MRI-guided laser therapy) used retrospective registry data for the main effect parameters, since they all were reimbursed without evidence for patient-relevant outcomes from RCTs [39], [40], [42]. Good research practices for comparative effectiveness research recommend the use of causal inference methods to adjust for confounding and selection bias in studies of treatment effects using secondary data bases [74], [75]. There was no information whether causal inference methods have been used to adjust for potential bias [76]. None of these studies explicitly mentioned the use of the target trial approach, which was emerging over the last years, to minimize bias in observational studies [77], [78], [79].

### Limitations

Our systematic review has several limitations. First, we may have missed relevant studies. We only found those that indicated by one of our keywords that the assessment was performed at an early phase in development. Not all relevant studies explicitly describe the early stage of their assessment. In addition, our search code may have had restrictions in other aspects that led us to miss important studies. Second, we neither gathered all information in supplementary data for each publication nor did we contact the authors of the original studies to seek further information. We may therefore have missed specific features of the modeling approaches. On the other hand, the wide range of applications, devices and modeling approaches in the nineteen studies included in our review provide an important and useful overview of recent modeling in early HTA of medical devices published since the review of IJzerman et al. [28] was performed. Finally, it is still an open question to which degree a thorough HTA process can influence the acceptance and reimbursement of medical technologies and how HTA impacts implementation in clinical practice [80]. Our results could be used to follow up on reimbursement, market access and routine care implementation of the medical devices assessed in this review and may close this gap for decision makers and manufacturers of medical devices.

## Conclusion and recommendations

In-vitro diagnostic tests for personalized and targeted medicine have become a major field of application for early decision-analytic modeling studies in early HTA. In the included studies, modeling allows for exploring clinical applications and target populations for new test-based interventions. According to our results, health-economic assessment is one of the main goals of developing decision-analytic models. Elicitation of beliefs and knowledge from panels of experts is a helpful strategy to substitute for empirical data in early HTA. Modeling is especially useful to explore the clinical utility of new tests.

The main exploited feature of decision-analytic models included in our review is their flexibility in assessing uncertainty through deterministic and probabilistic sensitivity analysis, threshold analysis and scenario analysis. Most studies use modeling types familiar in regular HTA, such as decision tree and Markov models. Patient-level discrete event simulation and systems modeling were also found in personalized medicine and modeling of societal systems, as predicted by previous reviews. Timed automata is a new model approach applied in the context of HTA that may be used more frequently in personalized medicine studies in the future when dynamic system behavior is involved.

For future research, we recommend:

- a separate and explicit assessment of benefit and harms, as well as the benefit-harm tradeoff, before cost-effectiveness analysis is performed;
- an explicit analysis along the ROC curve for the optimization of the positivity criterion defining when a test or biomarker level is called “positive”, both for benefit-harm and cost-effectiveness analyses;
- performance of value-of-information analysis as a core part of early HTA in medical devices to guide future research;
- the use of causal inference methods and the target trial approach when using observational data to derive model parameters.

In line with previous publications, we emphasize the importance of modeling the complete uncertainty surrounding novel biomarker testing, even if data are lacking in early assessment. Modeling should include all uncertainties associated with testing, including inconclusive test results, the negative consequences of false test results and of wait times for test results, and the uncertainties of the association of test results with the underlying disease, prognosis, treatment response and clinical outcomes.

## Notes

### Funding

This research was funded by the German Agency for Health Technology Assessment at the German Institute for Medical Documentation and Information (DAHTA@DIMDI), an Institute of the German Federal Ministry of Health. The authors had complete and independent control over study design, analysis and interpretation of data, report writing, and publication, regardless of results.

### Competing interests

The authors declare that they have no competing interests. Beate Jahn and Uwe Siebert have been members of the ISPOR-SMDM Modeling Good Research Practices Task Force.

## Supplementary Material

Tables 3–7

## Figures and Tables

**Table 1:**
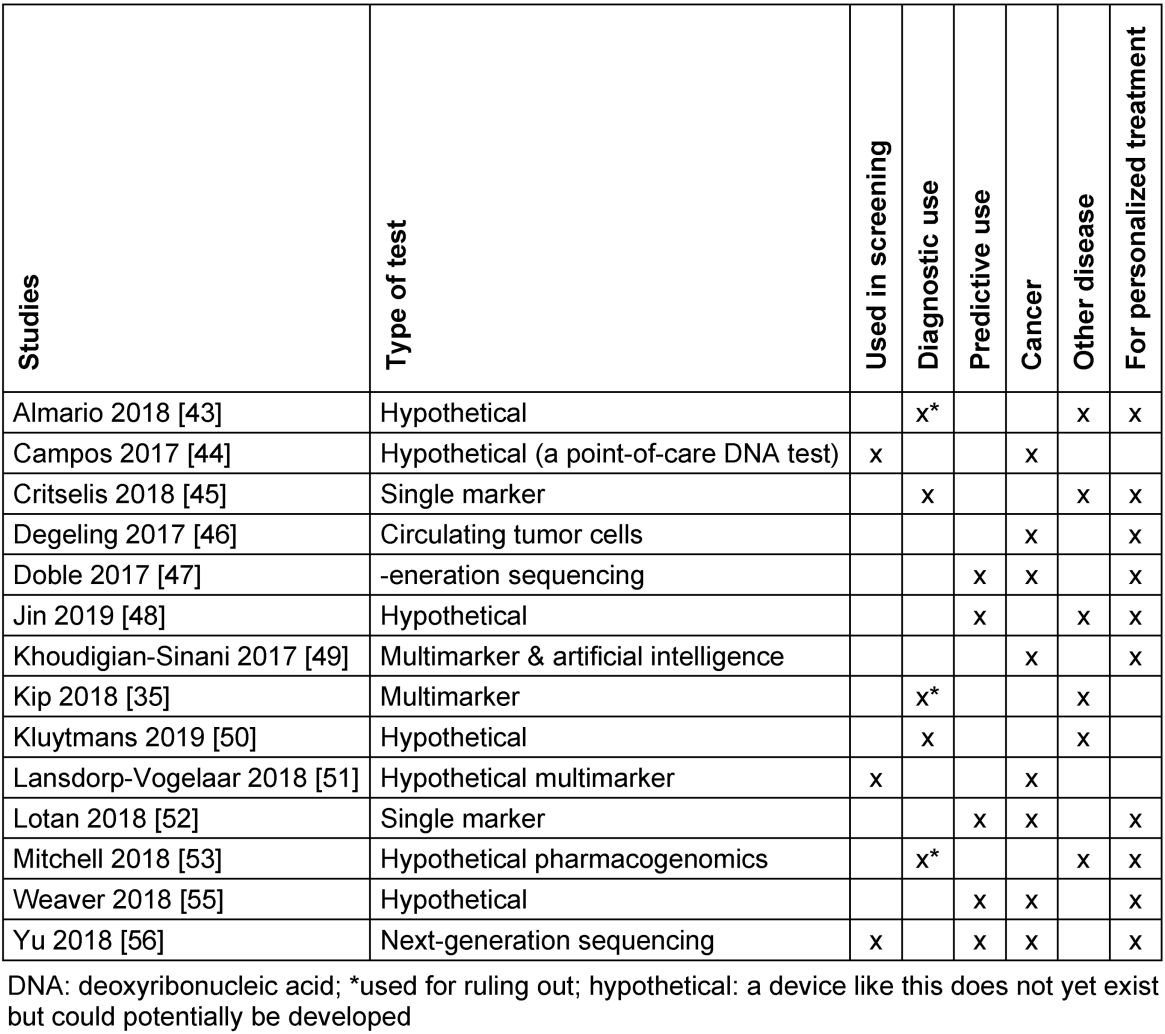
Overview of in-vitro diagnostic devices included in the review: type of test, setting, disease area of application, and whether the test aims at personalized treatment

**Table 2:**
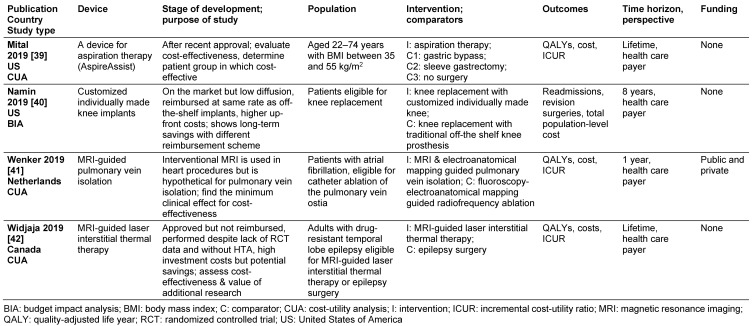
Therapeutic devices – device, stage of development and framework of the model
