# Supplementary material for: Decision-analytic modeling for early health technology assessment of medical devices – a scoping review
Source: Ger Med Sci. 2022 Dec 21;20:Doc11. doi: 10.3205/000313 (PMC9869403; doi:10.3205/000313)
Supplement: Tables 3–7 [file GMS-20-11-s-001.pdf]

Attachment

Table 3: Search code PubMed and related hits

Search	Query	Items found
#3	Search (#1 and #2) Filters: Full text; Publication date from 2017/05/01 to 2020/04/17; Humans; English	164
#2	Search ("decision-analysis"[All fields] or "decision analysis"[All fields] or "decision-analytic"[All fields] or "decision analytic"[All fields] or "decision tree"[All fields] or "markov model"[All fields] or "state-transition model"[All fields] or "discrete event simulation"[All fields] or "agent-based"[All fields] or "systems dynamics"[All fields] or "system dynamics"[All fields] or "dynamic modeling"[All fields] or "dynamic model"[All fields] or "microsimulation"[All fields] or "cohort simulation"[All fields])	33,711
#1	Search ((("early economic evaluation" or "early health technology assessment" or "early hta" or "early technology assessment" or "early assessment" or "early evaluation" or "early benefit assessment" or "early cost-effectiveness" or "early CEA" or "early health economic" or "headroom") OR ((hypothetical or "R&D" or "research and development" or approval or preapproval or emerging) AND (health technology assessment[mesh] or cost-benefit[mesh]))))	8,435

Table 4: Search code Embase and related hits

Search	Query	Items found
#5	#4 AND (2017:py OR 2018:py OR 2019:py OR 2020:py)	72
#4	#3 AND [english]/lim AND ([embase]/lim OR [embase classic]/lim) AND [humans]/lim AND [article]/lim	279
#3	#1 AND #2	598
#2	'decision-analysis' OR 'decision analysis' OR 'decision-analytic' OR 'decision analytic' OR 'decision tree' OR 'markov model' OR 'state-transition model' OR 'discrete event simulation' OR 'agent-based' OR 'systems dynamics' OR 'system dynamics' OR 'dynamic modeling' OR 'dynamic model' OR 'microsimulation' OR 'cohort simulation'	54,908
#1	'early economic evaluation' OR 'early health technology assessment' OR 'early hta' OR 'early technology assessment' OR 'early assessment' OR 'early evaluation' OR 'early benefit assessment' OR 'early benefit assessment' OR 'early cost-effectiveness' OR 'early cea' OR 'early health economic' OR 'headroom' OR ((hypothetical OR 'r&d' OR 'research and development'/exp OR 'research and development' OR approval OR preapproval OR emerging) AND (('health'/exp OR health) AND ('technology'/exp OR technology) AND ('assessment'/exp OR assessment) OR 'cost benefit'/exp OR 'cost benefit'))	16,866

Table 5: Therapeutic devices – model characteristics

Publication	Model Type	Effect data	Mechanism of effect	Cost data	Uncertainty; VoI Analysis	Elicitation Calibration Validation
**Mital 2019 [39]**	Decision tree & Markov model; cohort simulation	Weight loss, discontinuation, replacement, complication rates: from 4 years of post-market registry data, 200 patients, 47% of which discontinued over 4 years; for comparators: recent meta-analysis; disutility of new procedure: assumption	Lower weight loss but less procedural disutility than competitors; initial procedure less costly but affords regular replacement	Direct medical cost; initial procedure, replacement and complication cost: mostly based on assumptions	DSA for range of parameters but not disutilities; PSA (not clear which variables were included); no VoI	E: no<br>C: no<br>V: no
**Namin 2019 [40]**	Systems dynamics model for market adoption over time under reimbursement scenarios	90 day readmission rates based on retrospective study of 235 patients (authors consultants for ConforMIS) and on short term evidence on mechanical performance (equations to derive numbers not found); duration of rehabilitation: assumption; long-term evidence lacking; no RCT yet	Fewer revision surgeries (adverse events), shorter rehabilitation, higher cost of device; long-term savings not relevant for hospitals because reimbursement rate is a bundle payment for 69 days of tx; therefore exploration of reimbursement scenarios	Direct medical costs: cost of product (product, surgeons, operating room), recovery (in hospital, rehabilitation, at home), 90 day readmission, 3 year revision surgery; assumption: custom knee implant 25% more costly	Scenarios with 4 reimbursement schemes; PSA for wide range of many parameters; no VoI	E: no<br>C: for procedures over time<br>V: for procedures over time

Publication	Model Type	Effect data	Mechanism of effect	Cost data	Uncertainty; VoI Analysis	Elicitation Calibration Validation
**Wenker 2019 [41]**	Decision tree; cohort simulation	All parameters except the varied were assumed effect parameters from previous procedures at the local medical center (same for intervention and comparator)	Assumption: reduced recurrence rate for new intervention (assumed, varied), no difference in complications; direct procedural cost are higher for MRI-guided procedures, procedure time will be similar after short learning phase	Direct medical cost, no capital cost; additional MRI cost from diagnostic cardiac MRI at local medical center; other cost data from national data bases	Threshold analysis for minimum effect needed for cost-effectiveness; DSA for all model parameters<br>no VoI	E: no<br>C: no<br>V: no
**Widjaja 2019 [42]**	Decision-tree plus state transition model; microsimulation	Mortality after procedure: 1 retrospective study of 234 patients for intervention (some authors consultants for Medtronic); probability seizure free in first year after procedure: same study as above and published review; adverse events (complications, death during procedure), utilities from study on comparator	Inferior effect on seizures but lower risk of death after procedure; probabilities of subsequent treatment differ also, shorter hospital stay for new intervention, high investment cost for new intervention	Direct medical cost: capital costs and maintenance for system from manufacturer; other detailed resource use and costs from patient-level cost study and reimbursement tables	One way DSA for most parameters, threshold analyses, scenario analyses, PSA;<br>EVPI, EVPPI for the probabilities of events and progression after tx and for utilities	E: no<br>C: no<br>V: no

C: calibration; DSA: deterministic sensitivity analysis; E: elicitation; EVPI: expected value of perfect information; EVPPI: expected value of partial perfect information; MRI: magnetic resonance imaging; PSA: probabilistic sensitivity analysis; RCT: randomized controlled trial; tx: treatment; V: validation; VoI: value of information

Table 6: Diagnostic devices – device, stage of development and framework of the model

Publication Country, Study type	Device	Stage of development; Purpose of study	Population	Intervention; Comparators	Outcomes	Time horizon, Perspective	Funding
**Almario 2018 [43] US CUA, BIA**	Single biomarker test for diagnosis of irritable bowel syndrome with diarrhea; mainly for rule-out	Hypothetical; thresholds for accuracy and cost to achieve cost-effectiveness	Patients meeting Rome IV criteria for irritable bowel syndrome with diarrhea	I: Biomarker test first, immediate tx for positive result, upfront further diagnostic testing for negative results; C: usual care: immediate tx for all and further diagnostic testing for non-responders	QALYs, cost, ICUR, price and accuracy thresholds for cost-effective-ness, budget impact	1 year, health care payer	Manufacturer (Common-wealth Diagnostics International, Inc)
**Campos 2017 [44] 3 low and middle income countries CEA**	Diagnostic test; hypothetical point-of-care test (DNA test) for HPV (Example GeneXpert Omni)	Hypothetical, value of high investment evaluated; determine maximum cost for new test to be cost-effective	Women of the general population	I: HPV DNA screening (1 visit, test & tx at same visit) 3 times in a woman's lifetime at ages 30, 35, 40; C: same screening schedule but 2 visits, 1 for test, 1 for tx	Lifetime risk of cancer, LYs, lifetime cost, INMB, ICER	Lifetime, society	Bill & Melinda Gates Foundation (private foundation)

Publication Country, Study type	Device	Stage of development; Purpose of study	Population	Intervention; Comparators	Outcomes	Time horizon, Perspective	Funding
**Critselis 2018 [45] Europe CUA**	Single biomarker (CKD273 urinary peptide classifier); diagnostic test for early kidney disease in diabetes	Recently developed, prognostic ability of marker was shown, no clinical application yet; assess potential value of biomarker guided tx, find target population for cost-effectiveness	Diabetic patients, age 50; additional: high risk group with at least one more risk factor; low risk group without other risk factors	I: Annual screening with new biomarker and intensified tx if positive;<br>C: annual screening with SOC (urinary albumin excretion) and intensified tx if positive	QALYs, cost, ICUR	Lifetime (=40 years), health care payer	Public
**Degeling 2017 [46] Netherlands CUA**	Test for response monitoring; circulating tumor cells as response marker for guiding tx of prostate cancer	Approved for disease monitoring but first trial as response marker only recently started; simulation to fill data gap	Patients with metastatic castration-resistant prostate cancer	I: Response monitoring by circulating tumor cells;<br>C: Response monitoring by PSA and bone scan (SOC)	QALYs, cost, ICUR, NHB, NMB	Lifetime, health care payer	Public
**Doble 2017 [47] Australia CUA**	Predictive multiplex targeted sequencing pharmacogenomics (PGx) test (general) for response to 4th line lung cancer tx	Test accuracy data from study on UW-OncoPlex, a next generation sequencing assay (can be ordered from the lab); early value assessment in an iterative process along developments in PGx testing	Patients with lung cancer, eligible for 4th line tx	I: Multiplex targeted sequencing and tx only for positive & actionable;<br>C1: no testing and chemotherapy,<br>C2: no testing and supportive therapy	ICUR, LYs, QALYs, cost, EVPI, EVPPI	10 years, health care payer	Public, academia

Publication Country, Study type	Device	Stage of development; Purpose of study	Population	Intervention; Comparators	Outcomes	Time horizon, Perspective	Funding
**Jin 2019 [48] UK CUA**	Predictive test for response to 2nd line psychotic tx for patients with schizophrenia	Hypothetical; find accuracy and price for cost-effectiveness	Patients with schizophrenia who failed a first-line antipsychotic	I: New test and 2nd line antipsychotic if test is positive, if negative immediate clozapine;<br>C: 2nd line antipsychotic for all, clozapine only after this fails	QALYs, cost, increment al cost savings & QALYs	Lifetime, health care payer & society	Public
**Khoudigian-Sinani 2017 [49] Canada CEA**	Diagnostic test (multimarker & artificial intelligence for predicting risk of oral cancer), “Straticyte™”	On the market, not reimbursed by public payers; CEA should inform manufacturer, healthcare system, and individual patient whether investing in this product is worthwhile	Age >30, biopsy for oral cancer taken	I: Biomarker test + histology, excision when high risk;<br>C: histology only, excision when high risk	Cancer cases avoided, total cost, ICER	5 years, private payer and patient	MITACS Accelerate Program (Government and Proteocyte Diagnostic Inc)
**Kip 2018 [35] Netherlands CEA**	Diagnostic test; combination of 3 biomarkers for rule-out of non-ST elevation myocardial infarction (NSTEMI)	Type of test, not one specific product, test type available, triple test hypothetical; eliciting expert opinion on clinical utility of a new test	Patients with suspected NSTEMI in the emergency room	I1: new test alone at admission (t0) only,<br>I2: new test at t0 and high-sensitivity troponin (hsTn) test after 2 hours (t2),<br>I3: new test at t0 and hsTn at t2 and t6;<br>C: hsTn at t0, t2, t6	Time to discharge, cost, ICER (negative)	6 hours; hospital	No information found
**Kluytmans 2019 [50] Netherlands CUA**	Diagnostic biomarker test for primary aldosteronism, hypothetical	Hypothetical; find accuracy and price for cost-effectiveness	Age=40, resistant hypertension	Hypothetical new test; SOC: aldosterone-to-renin ratio	QALYs, costs, CE thresholds for price, accuracy	Lifetime/ 10 years/ 20 years, society	None declared

Publication Country, Study type	Device	Stage of development; Purpose of study	Population	Intervention; Comparators	Outcomes	Time horizon, Perspective	Funding
**Lansdorp-Vogelaar 2018 [51] Netherlands CEA**	Biomarker assay for colorectal cancer screening	Hypothetical; find accuracy and price for cost-effectiveness	Age 55–75, average risk for colorectal cancer	I: 84 screening strategies (start age, stop age, intervals) using a new test (35 different sets of test characteristics);<br>C: optimal fecal immunochemical test screening strategy as determined in previous study	LYs, cost per partici-pant, ICER; threshold price for test with certain character-istics	Lifetime, modified societal perspective (includes direct costs and patient time costs)	Not reported
**Lotan 2018 [52] US CEA**	Predictive single biomarker test for guiding neoadjuvant chemotherapy for muscle-invasive bladder cancer	Tests like this are on the market, some initial data, prognostic value for response shown for some; assess the cost-effectiveness, create a basis for incorporating biomarkers into clinical decision making	Patients with muscle-invasive bladder cancer, eligible for cisplatin-based neoadjuvant chemotherapy	I: Biomarker-guided NAC followed by radical cystectomie (RC);<br>C1: unselected neoadjuvant chemotherapy followed by RC;<br>C2: RC alone	LYs, 5 year survival, costs, ICER	5 years, health care payer	Public
**Mitchell 2018 [53] Canada CUA**	Diagnostic pharmacogenomics (PGx) test for rule-out of myopathy	Hypothetical; find accuracy and price for cost-effectiveness	Age >65, history of myocardial infarction or stroke, on statins, with muscle pain	I: PGx test for true myopathy and continuation on statins for negative result;<br>C1: no test and immediate stop of statin use;<br>C2 (sensitivity analysis): SOC test	ICUR, QALYs, cost	Lifetime, health care payer	Genome Canada, Genome Quebec (Public-private partnership)

Publication Country, Study type	Device	Stage of development; Purpose of study	Population	Intervention; Comparators	Outcomes	Time horizon, Perspective	Funding
**Terjesen 2017 [54]**<br>**US**<br>**CEA**	Single-use flexible video bronchoscope (Ambu[R] aScope[TM] 3, Monitor Ambu[R] aView[TM])	On the market; assess cost-effectiveness	Patients eligible for bronchoscopy	I: Bronchoscopy with single use device;<br>C: bronchoscopy with re-usable device	Avoided infections, incremental cost per bronchoscopy, ICER	1 year, health care payer	None
**Weaver 2018 [55]**<br>**US**<br>**Effective-ness analysis**	Predictive test for triage between two tx options in advanced stage epithelial ovarian cancer	Hypothetical; evaluate potential benefit for patients	Patients with newly diagnosed stage IIIC epithelial ovarian cancer	I: Test guided tx with either primary cytoreductice surgery or neoadjuvant chemotherapy and interval cytoreductive surgery;<br>C: triage and SOC tx	LYs	Lifetime, not applicable	None
**Yu 2018 [56]**<br>**US**<br>**BIA**	Predictive next generation sequencing test to guide first-line therapy in lung cancer	Type of test, not one specific product, increasingly available on the market but expensive; inform reimbursement decisions for next generation sequencing tests	Newly diagnosed patients with non-squamous advanced non-small cell lung cancer undergoing gene testing	I: next generation sequencing testing and targeted treatment;<br>C: single gene testing and targeted treatment	Budget impact	5 years, health care payer	Thermo Fisher Scientific (private)

BIA: budget impact analysis; C: comparator; CEA: cost-effectiveness analysis; CUA: cost-utility analysis; EVPI: expected value of perfect information; EVPPI: expected value of partial perfect information; I: intervention; ICER: incremental cost-effectiveness ratio; ICUR: incremental cost-utility ratio; LY: life year; NHB: net health benefit; QALY: quality-adjusted life year; SOC: standard of care; tx: treatment; UK: United Kingdom of Great Britain and Northern Ireland; US: United States of America; y: year

Table 7: Diagnostic devices – model characteristics

Publication	Model Type	Effect data	Mechanism of effect	Cost data	Uncertainty VoI	Elicitation Calibration Validation
**Almario 2018 [43]**	Decision tree	Test accuracy is assumed and varied; probabilities and utilities from several published studies (online appendix)	Rule-out: biomarker true negative results lead to earlier detection of other organic disease; biomarker false positive results lead to longer failed tx before detection of other organic disease	Reimbursement cost from public databases	PSA; no VoI	E: no<br>C: no<br>V: no
**Campos 2017 [44]**	Decision tree & state-transition model; individual level simulation	Test performance and effect of 1-visit testing from multi-site demonstration project (assumed same as for SOC test); screening coverage assumed 100%; loss to follow-up was assumed and varied	Avoidance of loss to follow-up between 2 screening visits; cost savings through 1-visit strategy compared to 2-visit strategy	Including indirect cost; from multi-site demonstration project	PSA; no VoI	E: no<br>C: natural history model calibrated previously<br>V: no
**Critselis 2018 [45]**	Markov model	Test accuracy and association with renal disease shown in studies; effect of intensified tx based on unspecified "reports"; SOC: UKPDS study	Higher sensitivity, specificity for new biomarker compared to SOC, more patients receive intensive hypertensive tx, fewer FP too; more costly	Retail price for test; resource use and payer cost: publications from European countries	DSA for many parameters; scenario analyses for target populations and for larger screening intervals; no VoI	E: "empirical reports" (expert opinion?) for effect of modified tx<br>C: no<br>V: internal & external for the SOC branch

Publication	Model Type	Effect data	Mechanism of effect	Cost data	Uncertainty VoI	Elicitation Calibration Validation
**Degeling 2017 [46]**	Timed automata & discrete event simulation, micro-simulation	Test accuracy and relation of cell count to survival from one published study; no RCT for circulating tumor cells as a response marker yet	Earlier tx switching from unsuccessful tx to next option leads to increased QoL; diagnostic performance of each test was considered; negative consequences for false positive results are not described; pathway includes: repetition of test according to guidelines, physician adherence to guidelines, tx interruptions not related to progression, survival based on individual patient history	Circulating tumor cell enumeration: expert opinion; other cost: literature, reimbursement tables	DSA for a large number of parameters; no VoI	E: physician adherence to guidelines and cost of test from experts' opinion<br>C: no<br>V: face validity, internal & cross validation; no data for external validation

Publication	Model Type	Effect data	Mechanism of effect	Cost data	Uncertainty VoI	Elicitation Calibration Validation
**Doble 2017 [47]**	Decision tree and Markov model	One study, reporting sensitivity & specificity of multiplex targeted sequencing panels in general in detecting any genomic alterations; tx effects: no effect data available on targeted tx with specific single alterations; therefore average tx effect for any targeted tx; all clinical transitions derived from published studies using a few assumptions	Benefit: some patients receive targeted therapy and benefit from higher response rate compared to standard tx; testing uncertainties considered: insufficient biopsy samples, test may not be successful, limited test accuracy, alterations may not be actionable; mortality during 4 week testing phase was considered; targeted tx starts at week five after successful test phase; start time of alternative tx during unsuccessful test phase was considered; adverse events from biopsy considered	Direct medical cost; sources: reimbursement rates, literature review, similar tests	DSAs for many parameters, scenario analysis for potential technological advances in the future;<br>EVPI, EVPPI	E: no<br>C: no<br>V: no
**Jin 2019 [48]**	Markov model	Test sensitivity and specificity are assumed and varied in full range, other data from published studies	Patients testing positive with the new test spend less time unnecessarily on clozapine, which has stronger adverse events than other antipsychotics	Price of test assumed and varied, guided by more expensive types of test; other cost: national data bases and published studies	One-way and two-way DSA;<br>threshold analysis, PSA;<br>no VoI	E: no<br>C: no<br>V: internal & face validation

Publication	Model Type	Effect data	Mechanism of effect	Cost data	Uncertainty VoI	Elicitation Calibration Validation
**Khoudigian -Sinani 2017 [49]**	Decision tree	Some assumptions; evaluation of 107 cases of dysplasia, with up to 10 years of follow-up, biomarker test + histopathology demonstrated improvement in (PPV) and (NPV) value by 10% and 27%, respectively	Risk stratification by new test in addition to stratification by histology; treatment scenarios for each combination elicited from experts	Direct medical and non-medical costs, indirect costs	Scenarios, one-way and PSA; no VoI	E: scenario drafting & belief elicitation for tx change with new device<br>C: no<br>V: no
**Kip 2018 [35]**	Decision tree	Main effects of test on clinical decisions were elicited for several test sensitivities; specificity of individual markers published, combined specificity calculated; sensitivity assumed (3 different values); other data from published evidence	New test shortens time to rule-out of non-ST-elevation myocardial infarction (effect on discharge rate and interventions performed); cost savings	Test cost: individual test cost were summed for triple test; hospital cost: reimbursement data	DSA for all parameters, PSA, Scenario analysis; no VoI	E: probability of discharge & follow-up diagnostics at different levels of accuracy elicited from 10 experts in questionnaire<br>C: no<br>V: no
**Kluytmans 2019 [50]**	Diagnostic decision tree, Markov model	Assumption	New test assumed to be perfect; TP, FP, FN, TN for old test; Tx if test is positive	Direct medical costs	Multivariate threshold analysis, one-way and PSA; headroom for price with perfect accuracy; no VoI	E: expert opinion for some parameters<br>C: no<br>V: no

Publication	Model Type	Effect data	Mechanism of effect	Cost data	Uncertainty VoI	Elicitation Calibration Validation
**Lansdorp-Vogelaar 2018 [51]**	State-transition model, micro-simulation	Test characteristics were assumed and varied, other parameters were available from previous models	Through sensitivity per person and per type of lesion and specificity	Direct medical costs, patient time cost, test cost assumed and varied; other costs updated from previous model	Range of scenario analyses, threshold analyses; no VoI	E: for some natural history parameters in previously published base models<br>C: some parameters in the base models were calibrated<br>V: no
**Lotan 2018 [52]**	Decision tree	A few data on mutation detection rates and response rates to treatment for 3 different biomarkers are available from small patient cohorts; combined to describe a general predictive single marker test; tests were not assumed to predict response perfectly	Depending on biomarker, fewer patients without response may get neoadjuvant chemotherapy and therefore avoid adverse effects without benefitting; more patients with response may get neoadjuvant chemotherapy and benefit	Test prices estimated from currently available tests	One-way DSA for range of parameters, 2-way DSA for test positivity and treatment response with positive result; no VoI	E: no<br>C: no<br>V: no
**Mitchell 2018 [53]**	Discrete event simulation, micro-simulation	Assumptions; percent of patients with true myopathy: assumption; “no test” assumed for SOC (stop statin if musculoskeletal pain); statin efficacy based on 1 RCT	Perfect sensitivity, specificity for PGx; more patients are staying on statin	Direct medical cost	Deterministic two-way SA for test accuracy, CE-threshold analysis, one-way DSAs, headroom: max price for CE threshold with ranges of test accuracy; no VoI	E: no<br>C: no<br>V: no

Publication	Model Type	Effect data	Mechanism of effect	Cost data	Uncertainty VoI	Elicitation Calibration Validation
**Terjesen 2017 [54]**	Decision tree	No evidence on infection risk found in a literature review, assumption of no risk of infection; comparator: risk of infection was elicited	Reduction of risk of infection to zero with new device; device cost higher but cost of one infection most important	Direct medical cost for device & adverse event; product price and average use; comparator: 5 published cost analyses; cost of infection: ventilator-assisted pneumonia assumed comparable	One- & two-way DSA, PSA; no VoI	E: Delphi-panel for risk of infection (8 experts completed 2 survey rounds)<br>C: no<br>V: no
**Weaver 2018 [55]**	Decision tree and state transition model, micro-simulation	Assumed test characteristics; other parameters: mainly a national cancer data base, survival after cytoreductive outcome from RCTs	Test-based triage leads to more complete resections; consequently to longer life expectancy	Not applicable	DSA for some parameters; no VoI	E: no<br>C: no<br>V: comparator arm against published data

Publication	Model Type	Effect data	Mechanism of effect	Cost data	Uncertainty VoI	Elicitation Calibration Validation
**Yu 2018 [56]**	Markov model for budget impact analysis	Rates of successful tests for next generation sequencing from one trial, tx effects from literature on single gene testing	Higher number of successful tests and patients on targeted therapy, rebiopsy and retesting considered if no success, two genes with targeted tx considered, two more in scenario analysis; clinical trial for mutations without proven targeted tx was considered; tx dosing based on patient characteristics	100% market uptake for next generation sequencing assumed, proportions of single gene tests according to current practice; reimbursement cost for tests, treatments, progression, palliative care	DSA (1-way) for broad range of parameters, scenario analyses; no VoI	E: expert opinion for enrollment rate in clinical trial, a few reimbursement rates<br>C: no<br>V: no

AE: adverse event; C: calibration; CE: cost-effective(ness); DSA: deterministic sensitivity analysis; E: elicitation; EVPI: expected value of perfect information; EVPPI: expected value of partial perfect information; FN: false negative; FP: false positive; NPV: negative predictive value; PPV: positive predictive value; PSA: probabilistic sensitivity analysis; QoL: Quality of life; RCT: randomized controlled trial; SOC: standard of care; TN: true negative; TP: true positive; tx: treatment; UKPDS: United Kingdom Prospective Diabetes Study; V: validation; VoI: value of information; y: year(s)
